# Masculinity, Perceived Vulnerability to COVID-19, and Adoption of Protective Behaviors

**DOI:** 10.1007/s12119-022-09991-5

**Published:** 2022-08-30

**Authors:** Michele Roccato, Maria Giuseppina Pacilli, Giovanni Orlando, Silvia Russo

**Affiliations:** 1 Department of Psychology, University of Torino, Via Verdi 10, 10124 Torino, Italy; 2 Department of Political Sciences, University of Perugia, Perugia, Italy

**Keywords:** COVID-19, Perceived vulnerability, Traditional masculinity ideology, Protective behaviors

## Abstract

Epidemiological data show that men and women have similar probabilities of contracting COVID-19. However, men with COVID-19 tend to have more severe outcomes than women. We performed two studies to analyze the associations between gender, adherence to traditional masculinity ideology, perceived vulnerability to COVID-19, and the adoption of protective behaviors against COVID-19. In Study 1 (quota sample of the Italian adult population, *N* = 1,142), we found no differences between men and women in terms of the perceived probability of contracting COVID-19. However, compared to women, men perceived themselves to be less likely to suffer severe consequences if falling ill. In Study 2 (Italian community sample, *N* = 305), a moderated mediation model showed that adherence to traditional masculinity ideology moderated the association between being man and the perceived severity of the consequences of COVID-19, which, in turn, showed negative associations with three protective behaviors against COVID-19. The article ends with a discussion of the strengths and limitations of this research.

## Introduction

As of July 2022, more than 500 million people worldwide have been infected with COVID-19 and more than 6 million have died due to the virus. Epidemiological data show men and women have similar probabilities of contracting COVID-19 (e.g., Chen et al., 2020; Zhao et al., 2020). However, compared to women, men with COVID-19 tend to have more severe outcomes (Peckham et al., 2020; Ueyama et al., 2020) and a higher mortality rate (Newman, 2021; Peckham et al., 2020). These results, which are consistent with those showing that women have longer life expectancies than men even in ‘normal times’ (Barford et al., 2006; Conti et al., 2003), are stable across contexts, recurring in France, Spain, Germany, Switzerland (Gebhard et al., 2020), China (Ahrenfeldt et al., 2021), Bangladesh (Al-Bari et al., 2021), and Canada (O’Brien et al., 2021), among other countries. The same pattern has been identified for Italy, where we conducted this research (Gebhardt et al., 2020).

Epidemiologists, virologists, and infectious disease specialists have attempted to explain these sex differences in the consequences of COVID-19 predominantly in terms of medical variables that differentiate men and women, such as differences in sex steroid hormones (Lipsa & Prabhu, 2021), levels of growth hormone (Lubrano et al., 2021), immune responses (Li & Li, 2021), genetic predispositions for contracting COVID-19 (Bartz et al., 2020), comorbidities (Salah & Mehta, 2020), and lifestyles (Abate et al., 2020). However, some researchers recommend employing a less reductionist approach to the topic, suggesting that the differences in consequences could depend on a complex interaction between biological, behavioral, environmental, and socioeconomic factors (Zarulli et al., 2018). For example, when ill, men are less predisposed toward help-seeking than women (Yousaf et al., 2015).

As Bridges and colleagues (2021) argued, cultural norms concerning gender have played a crucial role in the COVID-19 pandemic, both in terms of the macro level of analysis—because national decisions have been shaped by cultures of manhood—and on the micro level—how people perceive and experience the disease. Although scarce, preliminary research shows that such cultural norms also play a crucial role in the management of COVID-19. For instance, specifically in relation to protective behaviors against COVID-19, recent research has shown that stronger conformity to masculinity norms is associated with less positive attitudes toward protective equipment such as face masks (Mahalik et al., 2021; Palmer & Peterson, 2020).

### Men, Masculinity, and Health

In the literature on the relationship between sociodemographic characteristics and health, the gender differences show some of the most consistent associations with health outcomes (e.g., Mahalik et al., 2007). Overall, men do not live as long as women (Barford et al., 2006; Baum et al., 2021) and have higher mortality and morbidity rates across most diseases (Bilsker et al., 2010; Ng et al., 2014; White et al., 2011). Despite the fact that a variety of factors influence health and longevity (for example, biological vulnerabilities, access to health care, and so on), numerous health experts argue that a better understanding of men’s health requires a focus on social-psychological variables and modifiable health practices (APA, 2018).

Research has demonstrated the detrimental effects of gender inequality on women and their health, as well as the link between the gendered nature of men’s identities and their illness (Courtenay, 2000). The way society views the male body—more powerful and efficient and less vulnerable to disease than the female body—is strongly impacted by social expectations of masculinity. Masculinity refers to the social roles, behaviors, and meanings prescribed to men in a society at any given time, and it is produced both within societal institutions and through daily interactions (Connel, 1995; Kimmel, 2000). According to the theory of precarious manhood, masculinity necessitates constant public confirmation, because it is hard to achieve it and, equally, easy to lose it (Vandello & Bosson, 2013). Specifically, health-related beliefs and behaviors constitute a crucial means of proving masculinity, with low interest in one’s health representing a key marker of masculinity (Courtenay, 2003). Furthermore, since risk-taking behaviors are essential for validating masculinity, compared to women, men tend to take fewer precautions and are more likely to underestimate the health risks of their actions (Courtenay, 2000; Levant et al., 2009).

Consistent with this difference, medical and psychological research has shown that men are more likely than women to be overweight due to having less healthy eating habits (Gallus et al., 2015; Imamura et al., 2015), as well as to engage in coronary-prone behavior (Watkins et al., 1991). Furthermore, compared to women, men tend to consume more tobacco, alcohol, and drugs and report more problems consequent to this consumption, such as driving under the influence of alcohol or drugs (Geisner et al., 2004; Sher & Rutledge, 2007). Indeed, the proportion of men compared to women losing their lives in road accidents in Europe was 76% versus 24% in 2017 (European Commission, 2018). Research has also demonstrated the reluctance of men to consult doctors (Chapple & Ziebland, 2002), generalized low levels of health-seeking behavior (O’Brien et al., 2005), a lack of health knowledge (Ganong & Markowitz, 1987), and hesitancy to engage in health-promotion activities (Addis & Mahalik, 2003; Byrnes et al., 1999; Courtenay, 2003; Mahalik et al., 2006, 2007).

The reluctance men show in terms of the adoption of protective and preventive behaviors is particularly relevant in the context of the COVID-19 pandemic. The effective management of the pandemic required a strong and continuous effort by citizens, who, in the first months of the outbreak, had to accept stay-at-home directives, curfews, social distancing, obligatory face masks, and systematic hand hygiene. After nearly one year of the pandemic, at the end of 2020, a growing number of COVID-19 vaccines were made available, and the citizens were asked to accept being vaccinated against COVID-19. Voluntary acceptance of these requests, which were essential to protect both individual and collective health, was needed, as it would have been difficult for governments to make the actions mandatory (Cardenas et al., 2021). Unsurprisingly, a minority of citizens did not comply with such requests, and the NoVax movement even spread across countries (Barello et al., 2021; Salali & Uysal, 2020). According to the growing literature on this topic, the voluntary adoption of protective behaviors is hindered by social disadvantage, low perceived vulnerability to COVID-19, low trust in institutions and even in the scientists, systematic exposure to the social media, where fake news about COVID-19 was often spread, and populist orientations (Allington et al., 2021; Cesarotti et al., 2021; Roccato & Russo, 2021; Salali & Uysal, 2020).

### The Present Research

In the current study, we were interested in adopting a gendered perspective to examine the perceived vulnerability to COVID-19 and the adoption of protective behaviors against COVID-19. We conducted two studies. In Study 1, we examined the differences between men and women in terms of their perceived vulnerability to COVID-19. In Study 2, we tested a moderated mediation model with male gender as the exogenous variable, the adoption of protective behaviors against the virus as the outcome, perceived vulnerability to COVID-19 as the mediator, and adherence to traditional masculinity ideology as a moderator of the association between male gender and perceived vulnerability.

## Study 1

### Goals and Hypotheses

In this study, we analyzed the differences between men and women in terms of their perceived vulnerability to COVID-19, conceptualized as the perceived likelihood of COVID-19 infection and the perceived severity of COVID-19 consequences. We hypothesized that men would perceive lower infection likelihood (H1) and lower consequence severity (H2) than women.

### Participants and Procedure

We tested our hypotheses via a secondary analysis of data collected within the Consequences of COVID-19 (COCO) project (Cavazza et al., 2021). The COCO dataset includes data from a large quota sample of the Italian adult population, surveyed via email and stratified by sex, age, and geographic area of residence, which has been surveyed regularly since spring 2019. Although the data were collected to study the political opinions and behaviors of Italians, they include valuable information to answer our research questions. Specifically, we used data collected in October 2020, the beginning of the second wave of the pandemic. A total of 1,142 individuals (50.7% women, *M*_age_ = 50.44, *SD* = 14.38) had valid responses to all of the variables we measured.

## Measures

### Gender and Controls

Gender was coded as 1 (man) and 0 (woman). In the analyses, we controlled for age, years of education, having previously contracted COVID-19 (1 = yes, 0 = no), and perceived economic situation. We assessed the latter variable via a four-category item asking respondents whether their current income allowed them to live comfortably (4) or whether they found this very difficult (1) (cf. Roccato et al., 2021).

### Perceived Likelihood of COVID-19 Infection

The variable was measured on a scale from 1 ‘very unlikely’ to 4 ‘very likely’, using the following question: ‘How likely is it that you will contract COVID-19 in the next weeks?’.

### Perceived Severity of Health Consequences of COVID-19

The variable was measured on a scale from 1 (‘not at all worried’) to 4 (‘extremely worried’), using the following question: ‘How worried are you for about the health consequences of COVID-19 for yourself?’.

## Results

Table 1 shows the descriptive statistics for the variables we used and their bivariate correlations. Controlling for age, education, having previously contracted COVID-19, and perceived economic situation, we tested our hypotheses using two ordinary least squares (OLS) regression models designed to predict the perceived likelihood of COVID-19 infection and the perception of the severity of COVID-19 health consequences based on the respondents’ gender. In addition to a significant effect of age, indicating that older individuals perceived a lower likelihood of contracting COVID-19 but a greater severity of health consequences, the analyses showed a significant gender difference in perceptions of the severity of COVID-19 health consequences. Consistent with H2, compared to women, men reported lower levels of concern regarding the health consequences of the virus. However, contrary to H1, the association between gender and the perceived likelihood of COVID-19 infection was not significant (see Table 2).

**Table 1:** Study 1: Descriptive statistics for the variables we used and correlations between them

	*M*	*SD*	1.	2.	3.	4.	5.	6.	7.
1. Man	− 0.01	1.00	-	− 0.05	0.11***	0.09**	0.02	− 0.04	− 0.11***
2. Age	49.26	14.74		-	− 0.23***	− 0.02	− 0.05	− 0.07*	0.21***
3. Education	13.86	3.59			-	0.18***	0.03	0.06	− 0.04
4. Perceived economic situation	2.35	0.66				-	− 0.01	0.09**	− 0.03
5. Contracted COVID-19 (1 = yes)	0.06	0.24					-	0.05	− 0.09***
6. Perceived probability of COVID-19 infection	2.16	0.71						-	0.28***
7. Perceived severity of COVID-19 health consequences	3.03	0.90							-

*Note*. For being man and having vs. not having contracted COVID-19, the “mean” represents respectively the quota of men and of participants who contracted COVID-19. *** *p* < .001. ** *p <* .01. * *p* < .05

**Table 2:** Study 1: OLS regression models predicting the perceived probability of contracting COVID-19 and the gravity of its consequences

	Perceived probability of COVID-19 infection				Perceived severity of COVID-19 health consequences		
	Coeff.	*SE*	*p*		Coeff.	*SE*	*p*
Man	− 0.07	0.04	0.11		− 0.23	0.05	< 0.001
Age	− 0.00	0.00	0.01		0.01	0.00	< 0.001
Education	0.01	0.01	0.15		0.01	0.01	0.19
Perceived economic situation	− 0.02	0.03	0.48		− 0.06	0.04	0.08
Contracted COVID-19	0.11	0.09	0.23		− 0.26	0.11	0.02
*R* ^*2*^	0.01				0.07		

*Note*: The table reports unstandardized OLS regression coefficients

## Discussion

In this study, based on the literature on masculinity and health behaviors (Bridges et al., 2021; Courtenay, 2000), we examined the association between gender and both the perceived likelihood of COVID-19 infection and the severity of the health consequences of COVID-19. Consistent with epidemiological data showing that men and women have the same likelihood of contracting COVID-19 (Chen et al., 2020; Zhao et al., 2020), we found no differences in the perceived likelihood of developing COVID-19 between men and women. However, men perceived the severity of the consequences of infection to be lower compared to women. Indeed, admitting to being at risk of developing *severe* health consequences might threaten the perceived invulnerability of the male body more than admitting to only being at risk of contracting COVID-19. This is a particularly interesting finding because research has consistently shown that men are actually at a higher risk than women of suffering serious health consequences from COVID-19 infection (Newman, 2021; Peckham et al., 2020; Ueyama et al., 2020).

This study raises two new questions in this field. Indeed, research has shown that traditional masculinity ideology, defined as an individual’s internalization of cultural belief systems and attitudes toward masculinity (Levant, 1996), shapes expectations and beliefs about how men should think, feel, and behave in relation to aspects such as health issues. For example, men who adhere to traditional masculinity ideology consume more tobacco, alcohol, and illegal drugs (Courtenay et al., 2002; Pleck et al., 1994) and are also more likely to engage in risky sexual behavior, such as not using condoms (Courtenay, 1998). Therefore, the first question raised by the current findings pertains to whether the gender variability in the perceived risk of developing severe health consequences depends on the adherence to traditional masculinity ideology. Secondly, it would be beneficial to determine whether this perceived risk relates to the individual’s tendency to protect themselves (and, indirectly, their social network) from COVID-19. We address these open questions in Study 2.

## Study 2

### Goals and Hypotheses

Beyond replicating the results of Study 1 results with a new sample to check their robustness, in this study, we aimed to test a moderated mediation model linking gender, perceived vulnerability to COVID-19, adherence to traditional masculinity ideology, and the adoption of three protective behaviors against COVID-19. As in Study 1, we hypothesized that being a man would have a negative association with perceived health consequences of COVID-19 (H1). Additionally, based on the literature mentioned in the introduction section, we hypothesized that adherence to traditional masculinity ideology would moderate the relationship, expecting a significant relationship only among participants who adhere to such ideology (H2). Finally, we expected the perceived health consequences of COVID-19 to show a positive association with intention to get vaccinated against COVID-19 (H3a), social distancing (H3b), and hand hygiene (H3c).

### Participants and Procedure

Between April 2021 and May 2021, we performed an online survey with a community sample (*N* = 305, 64.9% women, *M*_age_ = 25.54, *SD* = 9.78) using snowball sampling. Specifically, the third author asked individuals in his social network to complete an online questionnaire and to forward the link to other people belonging to their social network and ask them to complete the questionnaire. The data were collected anonymously using Limesurvey.

## Measures

### Gender, Perceived Severity of Health Consequences of COVID-19, and Controls

Gender was coded as 1 (man) and 0 (woman). Perceived vulnerability and control variables were assessed using the same measures as in Study 1.

### Traditional Masculinity Ideology

To assess traditional masculinity ideology, we used the Male Role Norms Inventory–Short Form (MRNI-SF) by Levant and colleagues (2013), which consists of 21 items and scored on a scale ranging from 1 (‘strongly disagree’) to 7 (‘strongly agree’), Cronbach’s α = 0.92.

### Intention to Get Vaccinated against COVID-19

We asked our participants if they had been vaccinated against COVID-19. The possible responses were ‘Yes (even just the first dose)’, ‘No, but I will do it as soon as possible’, and ‘No, and I have no intention to do so’. We created a measure of intention to vaccinate against COVID-19 by contrasting those who were either vaccinated or intended to be vaccinated (1) with those who had no intention to be vaccinated (0).

### Social Distancing

We asked our participants how often they (a) distanced themselves from people outside the household (both strangers and friends and relatives), and (b) did not pay attention to social distancing on a scale from 1 (‘never’) to 7 (‘always’). Based on a correlation of *r* = .65, *p* < .001, between the two items, after recoding the second item we calculated a mean index of social distancing.

### Hand Hygiene

On a scale from 1 (‘never’) to 7 (‘always’), participants were asked to indicate how often they engaged in six hand hygiene behaviors. Examples of the items include ‘Wash your hands with soap when you go home’ and ‘Carry a bottle of sanitizing gel’. We computed a mean index of hand hygiene, with Cronbach’s α = 0.83.

## Results

Table 3 shows the descriptive statistics for the variables we used and their bivariate correlations. Consistent with H1, the preliminary analysis confirmed the negative association between being a man and perceived severity of the health consequences of COVID-19 identified in Study 1. Furthermore, consistent with Study 1, being a man was not associated with perceived probability of COVID-19 infection (see Table 4). In three moderated-mediated OLS regression models (performed using Process, Model 7), one for each behavioral outcome considered, we examined the associations between gender and the behavioral outcomes through the mediating variable of perceived severity of health consequences due to COVID-19. We also tested the moderating effect of traditional masculinity ideology (mean-centered) on the association between gender and perceived severity of COVID-19. The left-hand side of the model, which includes the moderated association between gender and perceived severity, is the same in all three models. In all the models, we controlled for age, years of education, having previously contracted COVID-19, and perceived economic situation.

**Table 3:** Study 2: Descriptive statistics for the variables we used and correlations among them

	*M*	*SD*	1.	2.	3.	4.	5.	6.	7.	8.	9.	10.
1. Man	0.35	0.48	-	− 0.02	− 0.00	− 0.00	0.04	− 0.08	− 0.17**	− 0.09	− 0.14*	− 0.34***
2. Age	25.54	9.78		-	0.08	− 0.07	0.04	− 0.05	0.20***	− 0.07	− 0.09	0.09
3. Education	15.69	2.88			-	− 0.04	− 0.01	0.02	0.04	0.04	0.02	0.03
4. Perceived economic situation	3.03	0.64				-	0.03	0.05	− 0.07	0.15-	− 0.02	− 0.05
5. Contracted COVID-19 (1 = yes)	0.17	0.38					-	0.14*	0.04	− 0.00	− 0.06	− 0.03
6. Perceived probability of COVID-19 infection	2.06	0.63						-	0.08	0.05	− 0.10	− 0.00
7. Perceived severity of COVID-19 health consequences	2.29	0.83							-	0.10	0.27***	0.23***
8. Intention to vaccinate	0.94	0.23								-	2.7***	0.28***
9. Social distance	4.79	1.37									-	0.52***
10. Hand hygiene	5.63	1.19										-

*Note*. For being man, having vs. not having contracted COVID-19 and the intention to vaccinate against COVID-19, the “mean” represents respectively the quota of men, of participants who contracted COVID-19 and of participants who have the intention to vaccinate. *** *p* < .001. ** *p <* .01. * *p* < .05

**Table 4:** Study 2: OLS regression models predicting the perceived probability of contracting COVID-19 and the gravity of its consequences

	Perceived probability of COVID-19 infection				Perceived severity of COVID-19 health consequences		
	Coeff.	*SE*	*p*		Coeff.	*SE*	*p*
Man	− 0.10	0.07	0.17		− 0.29	0.10	0.003
Age	− 0.00	0.00	0.48		0.02	0.00	0.001
Education	0.01	0.01	0.66		0.01	0.02	0.75
Perceived economic situation	0.05	0.06	0.41		− 0.08	0.07	0.28
Contracted COVID-19	− 0.23	0.09	0.02		0.09	0.12	0.45
*R* ^*2*^	0.03				0.07		

*Note*: The table reports unstandardized OLS regression coefficients

**Fig. 1:**
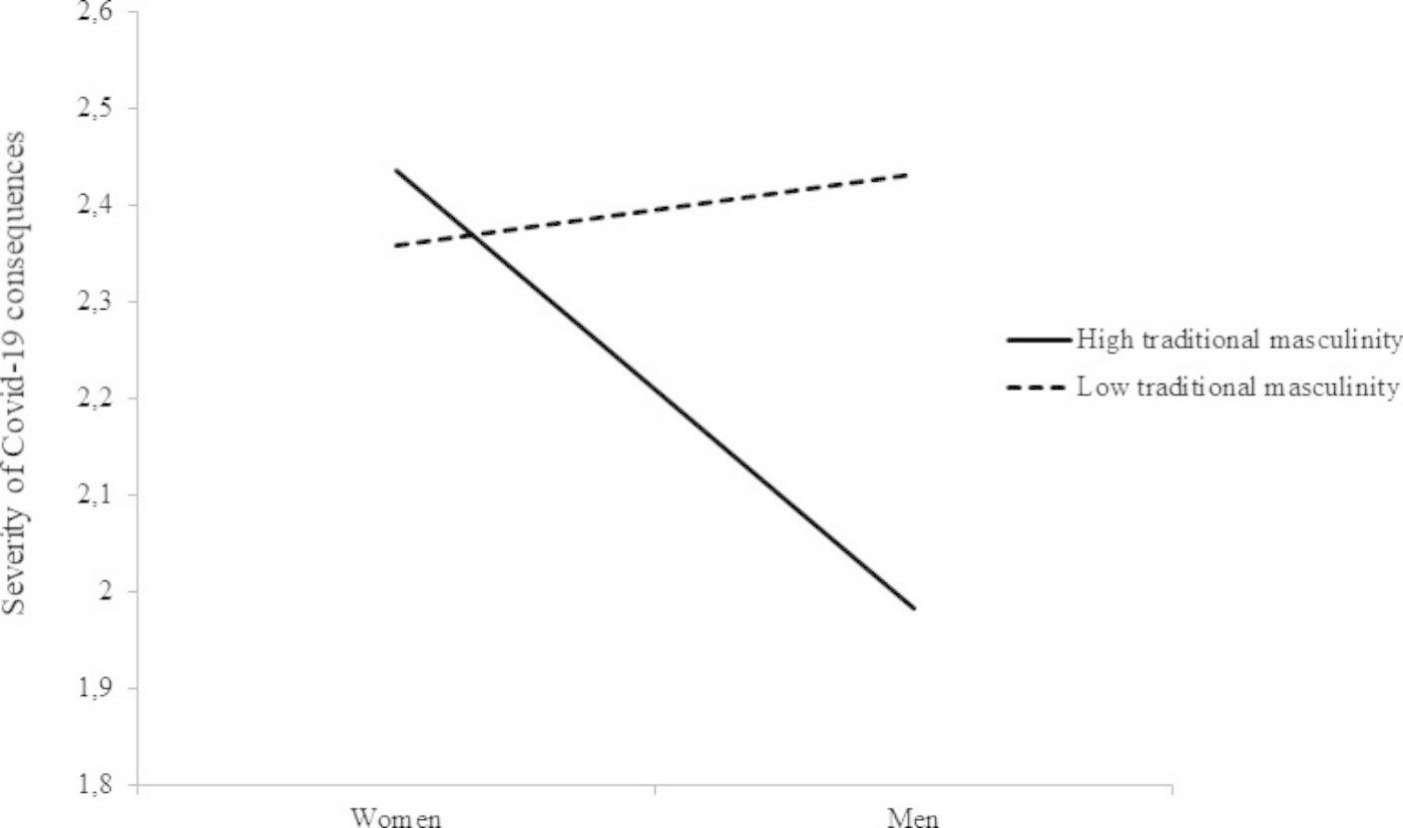
Association between being man and perception of the severity of COVID-19 health consequences as a function of the endorsement of traditional masculinity

Table 5 shows the results of these analyses. The direct relationship between gender and perceived severity of health consequences became non-significant when adherence to traditional masculinity ideology was included in the model, which demonstrates a significant relationship with it. In addition to the effect of age, which indicated that older people are more concerned about health outcomes than younger people, we found a significant interactive effect between gender and traditional masculinity ideology. A simple slope analysis revealed that, consistent with H2, men who endorsed traditional masculinity perceived the severity of health consequences to be lower than women (+ 1 *SD*, coeff. = − 0.45, *SE* = 0.14, *p* = .002, 95% CI − 0.73, −0.17), but this did not apply to men who rejected traditional masculinity (− 1 *SD*, coeff. = 0.07, *SE* = 0.14, *p* = .61, 95% CI − 0.21, 0.36). Figure 1 graphically depicts this interaction.

**Table 5:** Study 2: Moderated-mediated regression models predicting the intention to vaccinate, social distancing and hand hygiene

Outcome:	Intention to vaccinate				Social distance				Hand hygiene		
	Coeff.	*SE*	*p*		Coeff.	*SE*	*p*		Coeff.	*SE*	*P*
Perceived severity of consequences	0.67	0.35	0.05		0.41	0.10	< 0.001		0.24	0.08	0.003
Man	− 0.58	0.53	0.28		− 0.26	0.16	0.11		− 0.78	-14	< 0.001
Age	− 0.03	0.02	0.11		0.01	0.01	0.44		0.01	0.01	0.40
Education	0.05	0.08	0.54		0.00	0.03	0.91		0.00	0.02	0.90
Perceived economic situation	1.11	0.41	0.01		0.02	0.12	0.89		− 0.07	0.10	0.50
Contracted COVID-19	− 0.08	0.70	0.91		− 0.23	0.20	0.24		− 0.06	0.17	0.70
*R* ^*2*^	0.13				0.09				0.15		
Mediator:	Perceived severity of consequences										
	Coeff.	*SE*	*p*								
Traditional masculinity	0.05	0.10	0.64								
Man	− 0.19	0.10	0.07								
Traditional masculinity*man	− 0.33	0.12	0.01								
Age	0.02	0.00	< 0.001								
Education	0.00	0.02	0.96								
Perceived economic situation	− 0.08	0.07	0.29								
Contracted COVID-19	0.07	0.12	0.54								
*R* ^*2*^	0.11										

*Note*: The table reports unstandardized OLS regression coefficients, except for Intention to vaccinate where the coefficients are expressed in log-odds metric and Nagelkerke *R*^2^ is reported

Consistent with H3a, H3b, and H3c, the perception of the severity of COVID-19 health consequences was positively and significantly associated with all the behavioral outcomes (although the significance of the association with vaccination intention was right at the conventional *p* < .05 threshold). The conditional indirect effects analysis indicated that being a man had significant negative indirect associations with social distancing- and hand hygiene-related behaviors via the mediating variable of perceived severity of COVID-19 health consequences. These indirect effects were significant for those with high levels of endorsement of traditional masculinity only (social distancing: coeff. = −0.19, 95% CI − 0.35, −0.05; hand hygiene: coeff: −0.11, 95% CIs − 0.22, −0.02). The indirect effect of gender on vaccination intention did not reach statistical significance (coeff: −0.30, 95% CIs − 0.98, 0.11).^1^

## Discussion

Study 2 confirmed and complemented the findings of Study 1, showing that it is not being a man *per se*, but rather the combination of being a man and endorsing a traditional view of masculinity that is negatively related to severity perceptions and prevention behaviors in relation to COVID-19. We found that conforming to traditional masculinity not only affects men’s beliefs—in terms of lower perceived severity of the health consequences of COVID-19—but also reduces men’s protective behaviors against COVID-19.

## General Discussion

Research on gender differences has shown that health beliefs and behaviors are influenced by the endorsement of traditional notions of femininity and masculinity (Courtenay, 2000). Men, particularly those who conform to traditional masculine norms, are particularly at risk as they view concern for their health as a sign of weakness (Levant et al., 2009). From this sociocultural perspective, concern for one’s own health may be incompatible with the prescriptions of masculine toughness, pose an intolerable threat to a man’s virility, and, thus, make concern about illness a dangerous signal of weak masculinity. Advancing this area of inquiry in the specific context of the COVID-19 pandemic, our research consistently showed that men (Study 1) and those who endorse traditional masculinity ideology (Study 2) perceive themselves as less vulnerable to the negative health consequences of COVID-19. More importantly, we found that endorsing traditional masculinity is negatively associated with not only the perceived severity of COVID-19 consequences but also with the likelihood of taking crucial protective measures, such as in terms of hand hygiene, social distancing, and vaccination.

It is important to acknowledge some limitations of the studies. First, our measures for perceptions of the likelihood of COVID-19 infection and the severity of health consequences were single items. Nevertheless, the methodological literature shows that single items tend to perform similarly when a construct is easily imagined (Rossiter, 2002) and are even better than full scales for surveying general population samples, as they minimize inaccurate responses and response sets (Bergkvist & Rossiter, 2007). Second, we focused on certain protective behaviors (i.e., intention to vaccinate, social distancing, and hand hygiene) but lacked measures of other behavioral outcomes, such as the use of masks or avoidance of crowded places. Moreover, we used cross-sectional data, which are suboptimal for testing mediation models. However, the reverse causation order is highly unlikely in this model. Finally, the sample we used in Study 2 was not representative of the general population, since it was recruited using a snowball sampling procedure. However, this is not a serious issue, as we were interested in the relationships between variables and not in their univariate distribution. Additionally, the results from the preliminary analyses we performed in Study 2 were in line with those from Study 1, which was conducted using a quota sample of the general population.

Similar to other Western nations, Italian society is undergoing a transformation of traditional gender roles and masculinity due to the feminist movement, as well as societal transformations in terms of two-career families, gay and lesbian families, etc. (Ciccone, 2019; Tager & Good, 2005). Cross-cultural research on the acceptance of traditional masculinity has consistently demonstrated that Italians hold comparable levels of acceptance with individuals from other Western nations, such as the United States (Bosson et al. 2021; Tager & Good, 2005). However, future research could investigate cultural differences in how traditional views of masculinity affect COVID-19-related health management strategies. As Kimmel and colleagues highlighted (2005), men and masculinity are shaped by factors such as age, class, ethnicity, and racialization, among others, and only at the intersections of other social divisions and social differences does the gendering of men exist. Therefore, future replications of our research could examine how belonging to an ethnic or sexual minority may moderate the relationships we have examined (Jaspal, 2021).

Despite these limitations, our study also has certain strengths. Masculinity is a set of socially prescribed ideas relating to the meaning of being a man in a particular time and culture (Connel, 1995; Kimmel, 2000). In order to conform to societal expectations of masculinity, men frequently engage in behaviors that endanger their physical and mental health. By advancing the study of health and masculinity in the specific context of the COVID-19 pandemic, our research confirms that the male need to appear physically, mentally, and emotionally strong also has an impact on COVID-19 management. In addition, our study is one of the first to demonstrate how a social-psychological factor, specifically adherence to traditional masculinity ideology, can jeopardize efforts to combat COVID-19. Indeed, men’s poor health affects not only the quality of life of men but also that of women since, in heterosexual couples, the women usually take care of the unwell man. In addition to female partners, children are also affected by their parents’ illness, and, further to this, society as a whole bears the social and economic costs of men’s health problems. During a pandemic such as COVID-19, men’s health becomes even more important, and “men’s health matters for everyone” (Ragonese et al., 2019, p. 11). In order to contain the spread of the virus and limit the number of deaths, adherence to health and safety protocols is essential. In this context, our study is particularly relevant because it shows that cultural norms leading to traditional gender beliefs can be dangerous not only at an individual level but also at a collective level.

## Data Availability

Electronic copies of the anonymized raw data, related coding information, and all materials used to collect data will be made available upon request.

## Footnotes

1 Twenty-one parallel models, performed using the MRNI-SF single items instead of the whole scale, led to substantially analogous results, which are available from the corresponding author.
